# SQ House Dust Mite Sublingual Immunotherapy Tablet in Children With Allergic Asthma: A Randomised Phase III Trial

**DOI:** 10.1111/all.70073

**Published:** 2025-10-09

**Authors:** Graham Roberts, Jocelyne Just, Hendrik Nolte, Ole Holm Hels, Andrzej Emeryk, Carmen Vidal

**Affiliations:** ^1^ The David Hide Asthma and Allergy Centre St Mary's Hospital Newport Isle of Wight UK; ^2^ NIHR Biomedical Research Centre University Hospital Southampton NHS Foundation Trust Southampton UK; ^3^ University of Southampton Faculty of Medicine and University Hospital Southampton Southampton UK; ^4^ Unité d'Allergologie, Hôpital Américain de Paris Neuilly sur Seine France; ^5^ Sorbonne Université Paris France; ^6^ CRESS, Inserm, INRAE, HERA Team Université Paris Cité Paris France; ^7^ ALK‐Abelló A/S Hørsholm Denmark; ^8^ Department of Pulmonary Diseases and Children Rheumatology Medical University of Lublin Lublin Poland; ^9^ Servicio de Alergología Complejo Hospitalario Universitario de Santiago, Santiago de Compostela A Coruña Spain

**Keywords:** allergen immunotherapy, allergic asthma, children, house dust mite, sublingual immunotherapy

## Abstract

**Background:**

In children, house dust mite (HDM) sensitisation is a contributing factor for developing allergic asthma. HDM allergen immunotherapy has demonstrated efficacy and safety in adults with allergic asthma; however, evidence for its use in children is limited. MT‐11 evaluated the efficacy and safety of the SQ HDM sublingual immunotherapy (SLIT) tablet in children (5–17 years) with HDM allergic asthma.

**Methods:**

This phase III, randomised, double‐blind, placebo‐controlled trial randomised 533 children with a recent history of asthma exacerbations, despite treatment with inhaled corticosteroids and/or long‐acting beta‐agonists, to daily treatment with SQ HDM SLIT‐tablet or placebo for 24–30 months. The primary endpoint was the annualised rate of clinically relevant asthma exacerbations. Adverse events (AEs) were reported throughout the trial.

**Results:**

The rate ratio for the annualised rate of clinically relevant asthma exacerbations was 0.89 (95% CI: 0.60, 1.31), in favour of the SQ HDM‐SLIT tablet; superiority over placebo was not established. Most treatment‐related AEs (TRAEs) were of mild or moderate severity, and few subjects discontinued due to TRAEs (< 2%). The most common TRAEs were local application site reactions (oral pruritus, throat irritation, ear pruritus, and upper abdominal pain). There was no increased incidence of asthma‐related events, and no anaphylaxis or adrenaline use in the SQ HDM SLIT‐tablet group.

**Conclusion:**

As a result of the coronavirus disease 2019 pandemic, asthma exacerbation rates were much lower than expected, contributing to the primary endpoint not being met. The SQ HDM SLIT‐tablet was well tolerated in a paediatric population with inadequately controlled HDM allergic asthma.

**Trial Registration:**

Clinicaltrials.gov identifier: NCT03654976; EudraCT number: 2016‐004363‐39

## Graphical abstract

Phase III trial investigating the efficacy and safety of SQ HDM SLIT‐tablet in children (5–17 years) with inadequately controlled HDM allergic asthma. Novel paediatric data showing that the safety profile of SQ HDM SLIT‐tablet is similar to that established in adults. There was no increased risk of asthma‐related AEs, and few children discontinued treatment due to AEs.

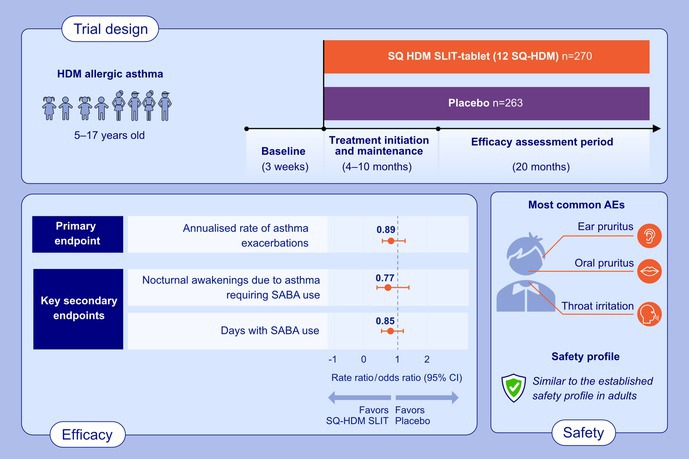

**Abbreviations**
AEs: adverse events
CI: confidence interval
HDM: house dust mite
SABA: short‐acting beta agonist
SLIT: sublingual immunotherapy

## 1 Background

Globally, house dust mite (HDM) allergens are contributing factors of persistent allergic rhinitis (AR) and allergic asthma [1, 2]. In children, early sensitisation to HDM is associated with an increased risk of developing HDM allergic asthma, resulting in a high prevalence of asthma among HDM‐sensitised children [2, 3]. Additionally, the likelihood of a child being hospitalised due to an asthma exacerbation increases when viral infections coincide with high exposure to the sensitising allergen (e.g., HDM) [4, 5].

Allergen immunotherapy (AIT) is currently the only treatment option that targets the underlying cause of allergic disease, providing sustained symptom relief for AR and reducing symptom‐relieving medication use [6, 7, 8]. AIT has the potential to protect against the progression from AR to allergic asthma [6, 7, 8]. However, AIT remains underused for asthma (and AR) treatment due to safety concerns [9, 10]. The SQ HDM sublingual immunotherapy (SLIT) tablet has demonstrated efficacy and safety in two clinical trials involving subjects with HDM allergic asthma—adults [11], and adults/adolescents [12]. This clinical evidence is reflected in the recommendations from the Global Initiative on Asthma (GINA), which state that HDM SLIT should be considered as an add‐on treatment to regular asthma therapy for steps 1–4 in adults (only if forced expiratory volume in 1 s [FEV_1_] is > 70% of predicted value) to reduce the risk of asthma exacerbation and improve asthma control [13]. The GINA recommendations are echoed by the European Academy of Allergy and Clinical Immunology (EAACI) guidelines, advocating for HDM SLIT tablet use as an add‐on treatment to regular asthma therapy in adults with controlled or partially controlled HDM allergic asthma [14]. In contrast to adults, evidence for the efficacy and safety of AIT in treating children and adolescents with HDM allergic asthma is relatively limited [14, 15]. An EAACI systematic review and meta‐analysis found that few studies in children have assessed the impact of AIT on asthma exacerbations, and the results are inconclusive [16]. To date, no safety concerns for AIT in children with AR and concomitant well controlled asthma have been identified [8]. However, AIT is not recommended in children with uncontrolled asthma due to limited evidence [8]. Consequently, there is a need to investigate the SQ HDM SLIT tablet in a paediatric population with inadequately controlled asthma.

This article presents the findings of a large phase III trial conducted to evaluate the efficacy and safety of the SQ HDM SLIT‐tablet in children (aged 5–17 years) with HDM allergic asthma.

## 2 Methods

### 2.1 Trial Design

MT‐11 was a randomised, double‐blind, placebo‐controlled, multi‐regional, phase III trial conducted at 68 sites in Bulgaria, Denmark, France, Germany, Hungary, Poland, Russia, Spain, United Kingdom, and the United States from February 2018 until May 2022. The trial design is shown in Figure S1. Subjects were randomised 1:1 to receive once‐daily treatment with SQ HDM SLIT‐tablet (12 SQ‐HDM dose) or placebo for 24–30 months as an add‐on to the subjects' own regular asthma controller medication (see Supporting Information, pages 5–6).

The trial was conducted in accordance with the Declaration of Helsinki and the International Conference for Harmonisation guidelines for Good Clinical Practice. The protocol, amendments, and other trial documentation were approved by the relevant ethics review boards and national regulatory authorities, according to local regulations. Written informed consent was obtained from the parent/caregiver of each child, and from the subject if they turned 18 years old during the trial. An independent Data Monitoring Committee monitored the trial to ensure subject safety and trial integrity.

### 2.2 Trial Population

The trial population included children aged 5–17 years (at randomisation) with physician‐diagnosed HDM allergic asthma of ≥ 1 year duration. Subjects were included if they had: a history of clinically relevant asthma exacerbations (≥ 3 in the past 2 years or ≥ 2 in the past year) or severe asthma exacerbations (≥ 1 in the past year) prior to randomisation despite treatment with a low daily dose of inhaled corticosteroids (ICS) plus long‐acting beta‐2 agonist (LABA), or a medium/high daily dose of ICS with or without LABA in the year before randomisation; FEV_1_ ≥ 70% of predicted value while on controller medication; at least one of the following within the 4 weeks before randomisation—daytime asthma symptoms more than twice per week, nocturnal awakenings due to asthma (requiring short‐acting beta‐2‐agonists [SABAs]), asthma symptoms more than twice per week requiring SABA treatment, or activity limitations due to asthma. A clinically relevant asthma exacerbation was defined as at least one of the following: doubling of ICS dose compared to controller medication; or systemic corticosteroids use for asthma symptom treatment for ≥ 3 days; or an emergency room visit due to asthma requiring systemic corticosteroids; or hospitalisation for > 12 h due to asthma requiring systemic corticosteroids. A severe exacerbation was defined using the aforementioned criteria without the criterion: doubling of ICS dose compared to controller medication. Additional inclusion criteria were: positive diagnostic tests (skin prick test and specific immunoglobulin type E [IgE] test) for HDM sensitisation to *Dermatophagoides pteronyssinus* and/or *Dermatophagoides farinae* at screening; a clinical history of HDM allergic rhinitis within the past year prior to randomisation (See Table S1 for the full list of inclusion and exclusion criteria).

### 2.3 Intervention Medication

The first tablet was administered at the trial site under medical supervision and the subject was observed for 30 min after intake. The SQ HDM SLIT‐tablet is an oral lyophilisate containing standardised allergen extract from two species of HDM (*D. pteronyssinus* and *D. farinae*). The dose tested was 12 SQ‐HDM. To maintain blinding, active, and placebo treatment were similar with regard to appearance, smell, and taste, and packaged identically. The aim for treatment compliance was ≥ 80% (defined as the proportion of tablets taken to the duration of exposure), assessed by counting the number of SLIT‐tablets remaining in the blister packaging at each visit.

Subjects were provided with asthma rescue medication, including reliever medication and treatment for asthma exacerbations. Throughout the trial, subjects were expected to continue with the same asthma controller treatment as before entering the trial (See S1, page 5).

### 2.4 Impact of the COVID‐19 Pandemic

Due to the coronavirus disease 2019 (COVID‐19) pandemic, screening and randomisation stopped early and therefore, fewer than the planned 600 subjects were enrolled. During the pandemic, the asthma exacerbation rate in the general population dropped, broadly attributed to the widespread adoption of virus containment measures (e.g., mask wearing, social distancing), which reduced the circulation of viruses known to trigger asthma exacerbations [17, 18, 19, 20]. Consequently, the recruitment of additional subjects with a recent history of asthma exacerbations was challenging. Therefore, the trial ended earlier than planned without any further recruitment (See Supporting Information, page 6).

### 2.5 Trial Endpoints

The primary efficacy endpoint was the annualised rate of clinically relevant asthma exacerbations, calculated as the number of exacerbations per year, per subject, during the 20‐month efficacy assessment period (Figure S1). A clinically relevant asthma exacerbation (as defined previously in the inclusion criteria and in Table S1) had to be medically confirmed by a trial investigator. Key secondary endpoints were evaluated every 4 months after randomisation and included the proportion of days with nocturnal awakenings due to asthma requiring SABA use, the proportion of days with SABA use (both recorded over 14 days in the electronic diary [eDiary] by the subject/caregiver), and assessment of lung function (percentage‐predicted FEV_1_). Additional secondary endpoints included: global evaluation of asthma at the end of the trial; total scores on the self‐reported Asthma Control Questionnaire (ACQ) (age ≥ 11 years) or the ACQ–Interviewer Administered (ACQ‐IA) (age 5–10 years) (hereafter, referred to as ACQ) evaluated every 4 months during the efficacy assessment period. Other endpoints included the change from baseline in immunological parameters (HDM‐specific IgE, IgG_4_, and IgE‐blocking factor [IgE‐BF]). Safety was evaluated by recording adverse events (AEs), assessing local allergic reactions and monitoring AEs of special interest (AESIs). During the first 28 days of treatment, solicited events of 15 prespecified local AEs were reported in an eDiary (based on recommendations of the World Allergy Organisation [WAO] [21]). AE solicitation is known to increase the reporting rate of local allergic reactions in clinical trials compared to unsolicited reporting of AEs [22]. (See Supporting Information, pages 6–7, for further descriptions of the trial endpoints.) In addition, asthma‐related events were analysed *post hoc* according to three subgroups of ACQ total score at baseline indicating the level of asthma control (ACQ ≤ 0.75 = well controlled; ACQ > 0.75 to < 1.5 = neither well nor poorly controlled; ACQ ≥ 1.5 = poorly controlled) [13].

### 2.6 Statistical Analysis

To ensure 90% power at the 5% significance level and to detect superiority of SQ HDM SLIT‐tablet treatment versus placebo, the trial planned to randomise 600 subjects (300 in each treatment arm), assuming an annual rate of 1.4 clinically relevant asthma exacerbations per subject in the placebo group (based on Lanier et al. [23]), a 30% dropout rate, and an efficacy assessment period of 20 months (86 weeks).

Efficacy analyses were conducted on the full analysis set (FAS; see Supporting Information, page 8, for the FAS definition). The primary endpoint was analysed using a negative binomial regression model with the number of asthma exacerbations as the response variable; treatment group, age group (< 12 years, ≥ 12 years), and region as fixed effects; and the logarithm of the time in the efficacy assessment period (in years) as an offset value. The between‐group comparison was performed using a Log Likelihood Ratio test derived from the negative binomial regression analysis; data are presented as rate ratio with Wald 95% confidence intervals (CIs). For the key secondary endpoints, the proportion of days with nocturnal awakenings and the proportion of days with SABA use were analysed using a marginal logistic regression model (presented as odds ratios with 95% CIs); the percentage‐predicted FEV_1_ was analysed using a mixed model for repeated measures (presented as absolute mean difference with 95% CIs). (See the Supporting Information, page 7–8, for the statistical analyses of other secondary endpoints). To adjust for multiplicity, hierarchical testing for superiority of the SQ HDM SLIT‐tablet over placebo was conducted for the primary and key secondary efficacy endpoints only (see Supporting Information, page 8); observed *p*‐values are presented for all other endpoints. Primary and key secondary efficacy endpoints were analysed using the primary estimand without imputations of missing data. Other secondary endpoints were analysed using observed data while on treatment. All statistical analyses were conducted by the trial sponsor using SAS version 9.4 (SAS Institute, Cary, NC). Safety analyses were conducted on the safety analysis set (see Supporting Information, page 8, for the safety analysis set definition) and are summarised descriptively. Asthma‐related events according to ACQ total score at baseline were analysed descriptively (*post hoc*); no formal statistical analysis was performed (See Supporting Information, pages 7–8, for further details of the statistical analyses).

## 3 Results

### 3.1 Population

Subject disposition is presented in Figure S2. A total of 533 subjects were randomised and treated with 12 SQ HDM SLIT‐tablet or placebo; 481 (90.2%) subjects completed treatment and 486 (91.2%) subjects completed the trial. Treatment compliance was high and similar between the two groups (SQ HDM SLIT‐tablet, 99.2%; placebo, 93.5%). Most subjects were treated with trial medication for ≥ 24 months (SQ HDM SLIT‐tablet, 88.1%; placebo, 90.9%). More treatment discontinuations due to AEs occurred in the SQ HDM SLIT‐tablet group (2.2%) than in the placebo group (0.4%). Baseline demographics and clinical characteristics were balanced across the two treatment groups (Table 1).

**TABLE 1:** Baseline demographics and clinical characteristics (FAS).

	Placebo (*N* = 260)	SQ HDM SLIT‐tablet (*N* = 264)
*Demographics*		
Age group, *n* (%)		
Children (5–11 years)	154 (59.2)	161 (61.0)
Adolescents (12–17 years)	106 (40.8)	103 (39.0)
Age (years), mean (SD)	10.6 (3.3)	10.5 (3.4)
Sex, *n* (%)^a^		
Female	93 (35.8)	85 (32.2)
Male	167 (64.2)	179 (67.8)
Race, *n* (%)		
White	251 (96.5)	253 (95.8)
Black/African American	5 (1.9)	6 (2.3)
Asian	0 (0.0)	1 (0.4)
Other	4 (1.5)	4 (1.5)
*Clinical characteristics*		
Duration of HDM allergic asthma (years), mean (SD)	4.5 (3.5)	4.4 (3.2)
History of asthma exacerbations, *n* (%)^b^		
≥ 1 severe asthma exacerbation in the past year	140 (53.8)	147 (55.7)
≥ 2 clinically relevant asthma exacerbations in the past year^b^, ^c^	97 (37.3)	97 (36.7)
≥ 3 clinically relevant asthma exacerbations in the past 2 years^b^, ^c^	15 (5.8)	13 (4.9)
Inclusion criterion for asthma history not met but included in trial^d^	8 (3.1)	7 (2.7)
Sensitisation, *n* (%)		
Monosensitisation (HDM only)	93 (35.8)	82 (31.1)
Polysensitisation (HDM and other allergens)	167 (64.2)	182 (68.9)
Baseline FEV_1_ (% predicted value), mean (SD)	97.3 (13.6)	96.2 (14.8)
Baseline ACQ total score	*N* = 257	*N* = 259
Mean (SD)	1.2 (0.7)	1.2 (0.8)
Median (P5%–P95%)	1.0 (0–2.4)	1.1 (0.1–2.6)
Min–max	0–4	0–4
Baseline ACQ total score according to level of asthma control, *n* (%)^e^	*N* = 263	*N* = 270
≤ 0.75	94 (35.7)	87 (32.2)
> 0.75 to < 1.5	77 (29.3)	88 (32.6)
≥ 1.5	89 (33.8)	90 (33.3)
Asthma severity (based on daily ICS dose at baseline), *n* (%)^f^		
Mild	5 (1.9)	5 (1.9)
Moderate	238 (91.5)	244 (92.4)
Severe	15 (5.8)	13 (4.9)

Abbreviations: ACQ, Asthma Control Questionnaire; FAS, full analysis set; FEV_1_, forced expiratory volume in 1 s; HDM, house dust mite; ICS, inhaled corticosteroid; P5%, 5% percentile; P95%, 95% percentile; SD, standard deviation; SLIT, sublingual immunotherapy.

^a^ Sex of subjects was reported by the investigator.

^b^ For the three criteria, each subject was counted only once and included in the first criterion that applied.

^c^ Included up to one asthma exacerbation that was not documented in medical records.

^d^ Out of the 15 subjects who did not fulfil the inclusion criterion for asthma history, four subjects had asthma exacerbations that occurred 1–2 months beyond the required year (exacerbations were recorded in medical records [*n* = 3] or no exacerbation was recorded but intake of systemic corticosteroids was recorded [*n* = 1]), and 11 subjects had asthma exacerbations that took place within the required timeframe but were not recorded in the medical record (verbal confirmation only).

^e^ *Post hoc* analysis using the safety analysis set; data were missing for three subjects in the placebo group and for five subjects in the SQ HDM SLIT‐tablet group.

^f^ Asthma severity was determined based on the daily ICS dose (low, medium, or high dose) at baseline, with pre‐defined thresholds for subjects 5–11 years old and ≥ 12 years of age (see Table S2). Asthma controller medication was not recorded for two subjects in each group, but the proportions were calculated based on the FAS.

### 3.2 Efficacy

**FIGURE 1:**
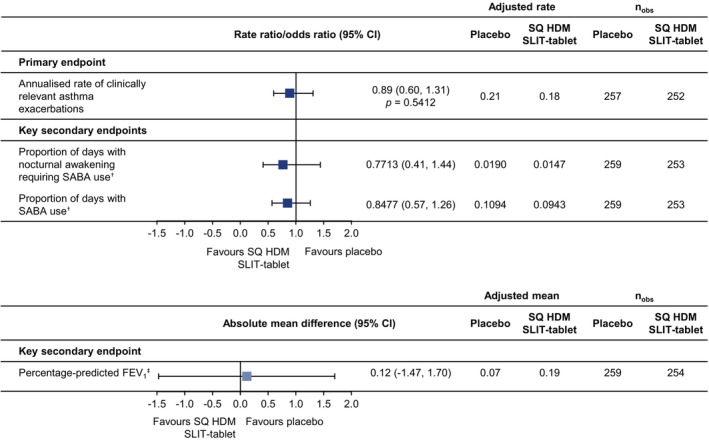
Primary and key secondary efficacy endpoint analysis (FAS, primary estimand). The key secondary endpoints could not be formally tested in the hierarchy and, therefore, *p*‐values are not presented. ^†^Measured using an eDiary over a 14‐day period, once every 4 months. ^‡^Measured every 4 months. CI, confidence interval; FAS, full analysis set; FEV_1_, forced expiratory volume in 1 s; HDM, house dust mite; n_obs_, number of subjects with observations contributing to the analysis; SABA, short‐acting beta‐2‐agonist; SLIT, sublingual immunotherapy.

The rate ratio for the annualised rate of clinically relevant asthma exacerbations (primary endpoint) was 0.89 (95% CI: 0.60, 1.31), numerically in favour of the SQ HDM‐SLIT tablet; superiority over placebo was not established (*p* = 0.5412) (Figure 1). According to the prespecified test hierarchy, formal statistical testing could not be conducted for the key secondary efficacy endpoints since the primary efficacy endpoint did not reach statistical significance; however, the point estimates were numerically in favour of the SQ HDM‐SLIT tablet (Figure 1).

The global evaluation of asthma at the end of the trial showed that subjects were more than twice as likely to have an improved outcome with SQ HDM SLIT‐tablet versus placebo (odds ratio = 2.26 [95% CI: 1.29, 3.96]; *p* = 0.004).

Asthma control (measured using the ACQ total score excluding FEV_1_) improved in both treatment groups, with no statistically significant differences between treatment groups at most timepoints (Figure S3).

**FIGURE 2:**
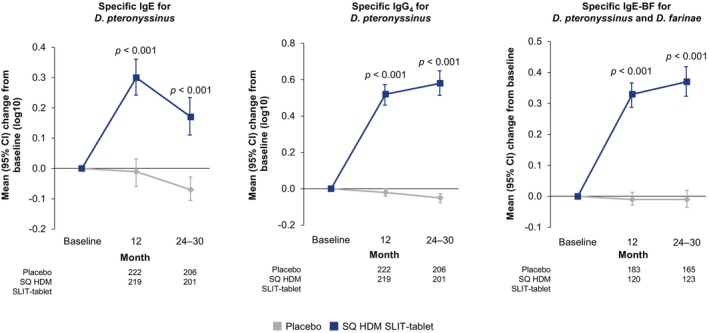
Changes from baseline in immunological parameters—HDM‐specific IgE, IgG_4_, and IgE‐BF (FAS). Numbers within the panel represent the number of subjects contributing to the adjusted mean. CI, confidence interval; FAS, full analysis set; HDM, house dust mite; IgE, immunoglobulin type E; IgE‐BF, immunoglobulin type E‐blocking factor; IgG_4_, immunoglobulin type G_4_; SLIT, sublingual immunotherapy.

Serum levels of HDM‐specific IgE and IgG_4_ for *D. pteronyssinus* increased in response to the SQ HDM SLIT‐tablet at 12 and 24–30 months after randomisation; they remained at, or below, baseline levels in the placebo group (Figure 2). A similar pattern was observed with specific IgE and IgG_4_ for *D. farinae* (Figure S4). Serum levels of IgE‐BF for *D. pteronyssinus* and *D. farinae* also increased in response to SQ HDM SLIT‐tablet, but not to placebo, at 12 and 24–30 months (Figure 2). The between‐group differences were statistically significant at all timepoints for the two HDM species across all immunological parameters (*p* < 0.001 for all comparisons).

### 3.3 Safety

Few subjects (< 2%) discontinued treatment due to treatment‐related AEs (TRAEs) (Table 2). The most common TRAEs in the SQ HDM SLIT‐tablet and placebo groups were local application site reactions (Table S3). The four most common TRAEs (oral pruritus, throat irritation, ear pruritus, upper abdominal pain) occurred shortly after treatment initiation (median time to onset: 1–7 days) and resolved within a few days (median duration: 1–2 days) (Table S4).

**TABLE 2:** Summary of TRAEs (safety analysis set).

	Placebo (*N* = 263)		SQ HDM SLIT‐tablet (*N* = 270)	
	*n* (%)	Events (%)	*n* (%)	Events (%)
All events	150 (57.0)	847 (100)	210 (77.8)	2491 (100)
Severity				
Mild	149 (56.7)	775 (91.5)	205 (75.9)	2288 (91.9)
Moderate	16 (6.1)	71 (8.4)	44 (16.3)	192 (7.7)
Severe	1 (0.4)^a^	1 (0.1)^a^	4 (1.5)^b^	11 (0.4)^b^
Severity by worst case^c^				
Mild	133 (50.6)	—	165 (61.1)	—
Moderate	16 (6.1)	—	41 (15.2)	—
Severe	1 (0.4)	—	4 (1.5)	—
Seriousness				
Not serious	150 (57.0)	847 (100)	210 (77.8)	2490 (99.96)
Serious	0 (0.0)	0 (0.0)	1 (0.4)	1 (0.04)
Outcome				
Recovered/resolved	150 (57.0)	844 (99.6)	210 (77.8)	2485 (99.8)
Recovered/resolved with sequelae	0 (0.0)	0 (0.0)	0 (0.0)	0 (0.0)
Not recovered/not resolved	2 (0.8)	3 (0.4)	1 (0.4)	4 (0.2)
Unknown	0 (0.0)	0 (0.0)	1 (0.4)	2 (0.1)
Changes to trial medication due to TRAE				
None	147 (55.9)	825 (97.4)	209 (77.4)	2413 (96.9)
Trial medication interrupted	12 (4.6)	21 (2.5)	12 (4.4)	65 (2.6)
Trial medication withdrawn	1 (0.4)	1 (0.1)	5 (1.9)	13 (0.5)
Discontinuation from trial due to TRAE	0 (0.0)	0 (0.0)	5 (1.9)	14 (0.6)

*Note:* Percentages were calculated from the total number of subjects in each group; within a particular category (e.g., severity, seriousness, outcome), some subjects reported several events, each with a different end result. An AE was considered treatment‐emergent if it started on or after the date the trial medication was first administered and/or no later than 7 days after the trial medication was last administered. TRAEs are TEAEs reported as ‘possibly’ related to trial medication by the investigator.

Abbreviations: AE, adverse event; HDM, house dust mite; SLIT, sublingual immunotherapy; TEAE, treatment‐emergent adverse event; TRAE, treatment‐related adverse event.

^a^ One subject experienced one event of atopic dermatitis (see Supporting Information, page 14, for narrative).

^b^ Collectively, four subjects experienced 11 severe TRAEs of lip pruritus, lip swelling, hypersensitivity, pharyngeal oedema, oral pruritus, ear pruritus, and/or throat irritation (see Supporting Information, pages 14–15, for narratives).

^c^ Each subject was counted only once and by worst severity among their reported events. Number of events is not applicable for ‘severity by worst case’.

No serious treatment‐related clinically relevant or severe asthma exacerbations were reported in either group. During the trial, no deaths, cases of anaphylaxis, or adrenaline use were reported, and no clinically relevant changes in laboratory parameters, vital signs, physical examinations, or lung function were observed in either treatment group. Few AESIs were reported during the trial (Table S5). Two subjects in the SQ HDM‐SLIT tablet group experienced a non‐serious, treatment‐emergent systemic allergic reaction reported by the investigator as hypersensitivity (see Supporting Information, pages 14–15, for narratives). Both events were assessed as possibly related to the trial medication, but neither case led to treatment discontinuation. In the SQ HDM‐SLIT tablet group, one subject experienced a non‐serious TRAE of severe local swelling/oedema of the mouth/throat and one subject experienced a serious TRAE of eosinophilic oesophagitis (see Supporting Information, pages 14–15, for narratives). In both cases, trial medication was discontinued, and the subject recovered.

**FIGURE 3:**
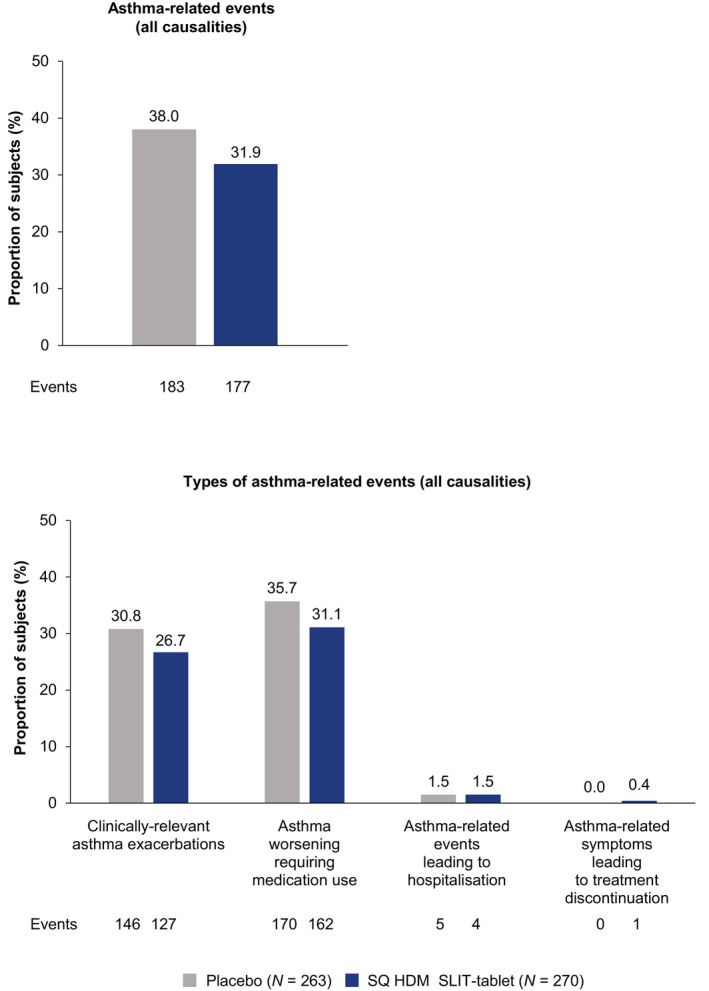
Asthma‐related events (safety analysis set). HDM, house dust mite; SLIT, sublingual immunotherapy.

The proportion of subjects with asthma‐related events (all causalities) was lower in the SQ HDM SLIT‐tablet group than in the placebo group (Figure 3A). The majority of asthma‐related events reported in the SQ HDM‐SLIT tablet group were non‐serious (*n* = 173/177; 97.7%) and most events were considered by the investigator to be unlikely related to trial medication (*n* = 171/177; 96.6%). When categorised by type of asthma‐related event, the proportion of subjects with events in the SQ HDM SLIT‐tablet group was lower or comparable to the placebo group (Figure 3B).

**FIGURE 4:**
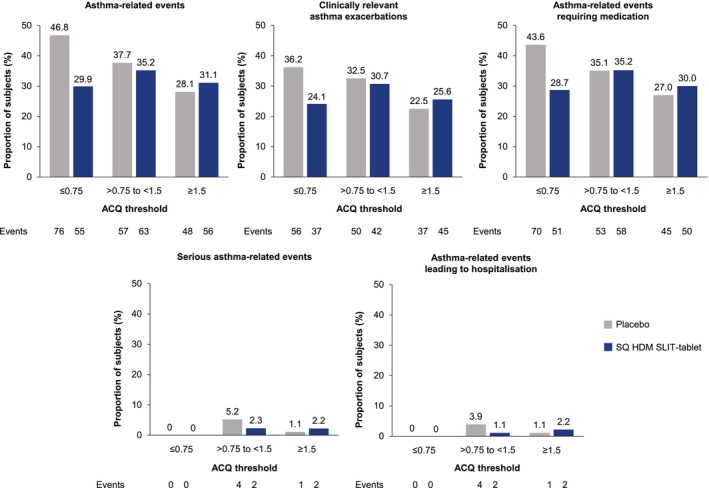
*Post hoc* analysis of asthma‐related events (regardless of causality) according to ACQ total score at baseline (safety analysis set). Panels A–E show all events reported irrespective of whether the investigator reported them as treatment‐related or not. The *post hoc* analysis of asthma‐related events according to asthma control at baseline assigned using ACQ total score thresholds from GINA: ACQ ≤ 0.75 = well controlled; ACQ > 0.75 to < 1.5 = neither well nor poorly controlled; ACQ ≥ 1.5 = poorly controlled. The overall number of subjects in each subgroup was: For placebo, ACQ ≤ 0.75 *n* = 94, ACQ > 0.75 to < 1.5 *n* = 77, ACQ ≥ 1.5 *n* = 89 (three subjects had missing ACQ data at baseline); for SQ HDM SLIT‐tablet, ACQ ≤ 0.75 *n* = 87, ACQ > 0.75 to < 1.5 *n* = 88, ACQ ≥ 1.5 *n* = 90 (five subjects had missing ACQ data at baseline). ACQ, Asthma Control Questionnaire; HDM, house dust mite; SLIT, sublingual immunotherapy.

The *post hoc* analysis of asthma‐related events according to asthma control at baseline (assigned using ACQ total score thresholds from GINA) demonstrated a lower or similar incidence of events with the SQ HDM SLIT‐tablet versus placebo across the three ACQ subgroups (Figure 4), indicating a good tolerability profile of the SQ HDM SLIT‐tablet irrespective of asthma control at baseline.

## 4 Discussion

MT‐11 contributes to our understanding of the efficacy and safety of the SQ HDM SLIT‐tablet in children (5–17 years) with inadequately controlled HDM allergic asthma. The trial demonstrated a low discontinuation rate over a 2‐year period. Overall, the SQ HDM SLIT‐tablet was well tolerated. The safety profile was comparable to the established safety profile in previous clinical trials that included adults with HDM allergic asthma [11, 12], and adults, adolescents, and children with HDM AR with or without asthma [24, 25, 26, 27]. There was no increased incidence of asthma‐related events in children with HDM allergic asthma receiving SQ HDM SLIT‐tablet—an important finding for this patient population. The incidence of asthma‐related events was generally lower with the SQ HDM SLIT‐tablet versus placebo. The annualised rate of clinically relevant asthma exacerbations (primary endpoint) was 11% lower with the SQ HDM SLIT‐tablet versus placebo; however, statistical significance over placebo was not established. Several factors likely contributed to this outcome.

The overall annualised rate of asthma exacerbations was low in both groups (0.21 with the SQ HDM SLIT‐tablet and 0.18 with placebo), which could largely be attributed to the approximately 67% decrease in asthma exacerbation rate during the COVID‐19 pandemic compared to the pre‐pandemic rate (see Supporting Information, page 6). Moreover, the exacerbation rate in the placebo group before the pandemic (0.39) was substantially lower than the expected rate of 1.4 described in the MT‐11 trial design based on the trial by Lanier et al.—the most comparable study to MT‐11 due to the inclusion of a paediatric population with inadequately controlled asthma despite medium‐dose or high‐dose ICS treatment, with or without other controller medication [23]. The rate observed in MT‐11 aligns more closely with data from subsequent trials in children with inadequately controlled asthma, where annualised asthma exacerbation rates of 0.43–0.87 were reported in the placebo group [28, 29, 30, 31]. This observation indicates that management of asthma symptoms in children has generally improved since the completion of the Lanier et al. trial [23, 28, 29, 30, 31], potentially due to reduced outdoor pollution, changes in healthcare utilisation, and improved asthma self‐management [18].

There were considerably lower levels of respiratory tract infections during the COVID‐19 pandemic, due to imposed social distancing measures and restricted social interaction [20, 32, 33]. This may have also impacted the primary efficacy assessment in MT‐11. Viral respiratory infections are a key trigger for asthma exacerbations in children; levels of respiratory syncytial virus and rhinovirus infections were lower during the COVID‐19 pandemic versus pre‐pandemic levels [33]. Consequently, there were many fewer hospital admissions for infections and asthma/wheeze than before the pandemic [20].

Further, asthma control in both treatment groups was found to improve during MT‐11, possibly due to the positive influence of participating in a clinical trial environment. Particularly during the COVID‐19 pandemic, children with asthma may have adhered more closely to their asthma controller medication, driven by heightened awareness of health risks associated with asthma and COVID‐19 [32]. Free access to reliever medication (SABA) and treatment for asthma exacerbations in MT‐11 may explain the relatively small difference in asthma‐related outcomes between treatment groups.

The MT‐11 trial design may have influenced the ability to detect a treatment effect on asthma outcomes (e.g., asthma exacerbations). Two previous clinical trials were successfully conducted in subjects with inadequately controlled HDM allergic asthma who received SQ HDM SLIT‐tablet: one trial in adults reported an improvement in asthma outcomes (including asthma exacerbations) [11], and the other trial in adults/adolescents demonstrated a reduction in ICS dose [12]. Subjects in these two trials were required to reduce their asthma controller medication during the efficacy assessment period [11, 12]. In MT‐11, where the target population included children, a reduction in controller medication was not permitted and may have limited the detection of a treatment effect. However, one Japanese trial in adults with HDM allergic asthma that included an ICS reduction period when assessing the asthma exacerbation rate following SQ HDM SLIT‐tablet treatment did not meet its primary endpoint [34]. These findings were attributed to factors including strict inclusion criteria for well‐controlled asthma, as defined by Japanese guidelines [35], and subjects reporting higher baseline doses of ICS and lower SABA use during the trial in contrast to European‐equivalent trials [34].

As immunotherapy is associated with specific changes in immunological markers [36], MT‐11 evaluated several immunological endpoints. Consistent with previous trials that have demonstrated the clinical effects of SQ HDM SLIT‐tablet trials on HDM AR or allergic asthma [11, 24, 25], changes in HDM‐specific IgE, IgG_4_, and IgE‐BF were observed in MT‐11 with the SQ HDM SLIT‐tablet, but not with placebo. The data demonstrate that the SQ HDM SLIT‐tablet effectively modulates the immunological response to HDM allergens.

Over the 2‐year trial period, there were few discontinuations due to TRAEs and no cases of anaphylactic reactions or adrenaline use. One case of eosinophilic oesophagitis occurred in MT‐11—the symptoms developed shortly after trial initiation (Day 6). The timing of the event suggests this was likely related to an underlying subclinical disease that was present but remained asymptomatic and undiagnosed prior to the start of the trial. No cases of eosinophilic oesophagitis have been reported in previous paediatric trials assessing SQ SLIT‐tablets [25, 37, 38].

A strength of MT‐11 was the long exposure of children to the SQ HDM SLIT‐tablet (~2 years), allowing for a robust assessment of safety and providing important data to meet the evidence gap for double‐blind, placebo‐controlled trials in children with inadequately controlled HDM allergic asthma. A limitation was the difference in trial design in comparison to adult asthma trials where corticosteroid tapering prevents direct comparisons against paediatric trials. Consequently, there remains a need to investigate the potential steroid‐sparing effect of the SQ HDM SLIT‐tablet in children with HDM allergic asthma in future trials. Another limitation may be the generalisability of the trial population, which was selected based on stringent inclusion and exclusion criteria, and may not reflect real‐life populations. This highlights the need for future real‐world studies that explore the effect of the SQ HDM SLIT‐tablet in children with allergic asthma.

In summary, MT‐11 did not meet the primary endpoint largely due to the lower‐than‐expected asthma exacerbation rate, which was severely impacted by the COVID‐19 pandemic. However, this trial has generated novel paediatric safety data. The safety profile of SQ HDM SLIT‐tablet in a paediatric population with inadequately controlled HDM allergic asthma was similar to the established safety profile in adults. Furthermore, results from MT‐11 supplement the findings from the previously published MT‐12 trial—a large, double‐blind, placebo‐controlled phase III trial, which demonstrated the clinical efficacy and safety of the SQ HDM SLIT‐tablet in 1460 children (5–11 years) with moderate‐to‐severe HDM AR/rhinoconjunctivitis with or without concomitant asthma [25]. The findings from the MT‐11 and MT‐12 trials provide important safety data to support decision‐making for HDM AIT use in a paediatric population with HDM respiratory allergy.

## Author Contributions

All authors contributed to data analysis and/or interpretation as well as to preparing and critically reviewing the manuscript. All authors had access to the trial data, reviewed the manuscript, revised the content, approved the final version for submission, and agree to be accountable for all aspects of the work. Ultimate responsibility for the opinions, conclusions, and data interpretation lies with the authors.

## Conflicts of Interest

G.R. was an investigator in the ALK‐Abelló Grazax Asthma Prevention (GAP) trial, a consultant/speaker for ALK‐Abelló A/S, a consultant for AstraZeneca, an author of the British Thoracic Society/Scottish Intercollegiate Guidelines Network (SIGN) guideline, Past President of the British Society for Allergy and Clinical Immunology (BSACI), and has received research funding from the National Institute for Health and Care Research (NIHR), National Institutes of Health (NIH), the EU, the Medical Research Council (MRC), and Action Medicine Research. A.E. has received lecture fees from ALK‐Abelló A/S. H.N. and O.H.H. are employees of ALK‐Abelló A/S. J.J. is a member of the ALK‐Abelló Paediatric Advisory Board and has received fees for lectures and consulting activities from ALK‐Abelló A/S, Stallergenes‐Greer, GSK, Novartis, AstraZeneca, and Zambon. C.V. has received funding for educational and research activities from ALK‐Abelló A/S, Allergy Therapeutics, AstraZeneca, GSK, HAL, Industry Roxal, Leti, and Stallergenes‐Greer.

## Supporting information


**Appendix S1:** all70073‐sup‐0001‐AppendixS1.docx.

## Data Availability

Further requests for data relating to the MT‐11 trial can be made through the ALK‐Abelló website, ‘Sharing our clinical trials data’ at: https://www.alk.net/our‐science/clinical‐data‐sharing.
