# Supplementary material for: SQ House Dust Mite Sublingual Immunotherapy Tablet in Children With Allergic Asthma: A Randomised Phase III Trial
Source: Allergy. 2025 Oct 9;80(12):3401–11. doi: 10.1111/all.70073 (PMC12666737; doi:10.1111/all.70073)
Supplement: Supplementary file 1 — Appendix S1: all70073‐sup‐0001‐AppendixS1.docx. [file ALL-80-3401-s001.docx]

**Supporting information**

**SQ house dust mite sublingual immunotherapy tablet in children with allergic asthma: a randomised phase III trial**

Graham Roberts,^1-3^ Jocelyne Just,^4,5,6^ Hendrik Nolte,^7^ Ole Holm Hels,^7^ Andrzej Emeryk,^8^ Carmen Vidal^9^

^1^The David Hide Asthma and Allergy Centre, St Mary’s Hospital, Newport, Isle of Wight, UK

^2^NIHR Biomedical Research Centre, University Hospital Southampton NHS Foundation Trust, Southampton, UK

^3^University of Southampton Faculty of Medicine and University Hospital Southampton, Southampton, UK

^4^Unité d’Allergologie, Hôpital Américain de Paris, Neuilly sur Seine, France,

^5^Sorbonne Université, Paris, France

^6^CRESS, Inserm, INRAE, HERA Team, Université Paris Cité, Paris, France

^7^ALK-Abelló A/S, Hørsholm, Denmark

^8^Department of Pulmonary Diseases and Children Rheumatology, Medical University of Lublin, Lublin, Poland

^9^Servicio de Alergología, Complejo Hospitalario Universitario de Santiago, Santiago de Compostela, A Coruña, Spain

**Methods**


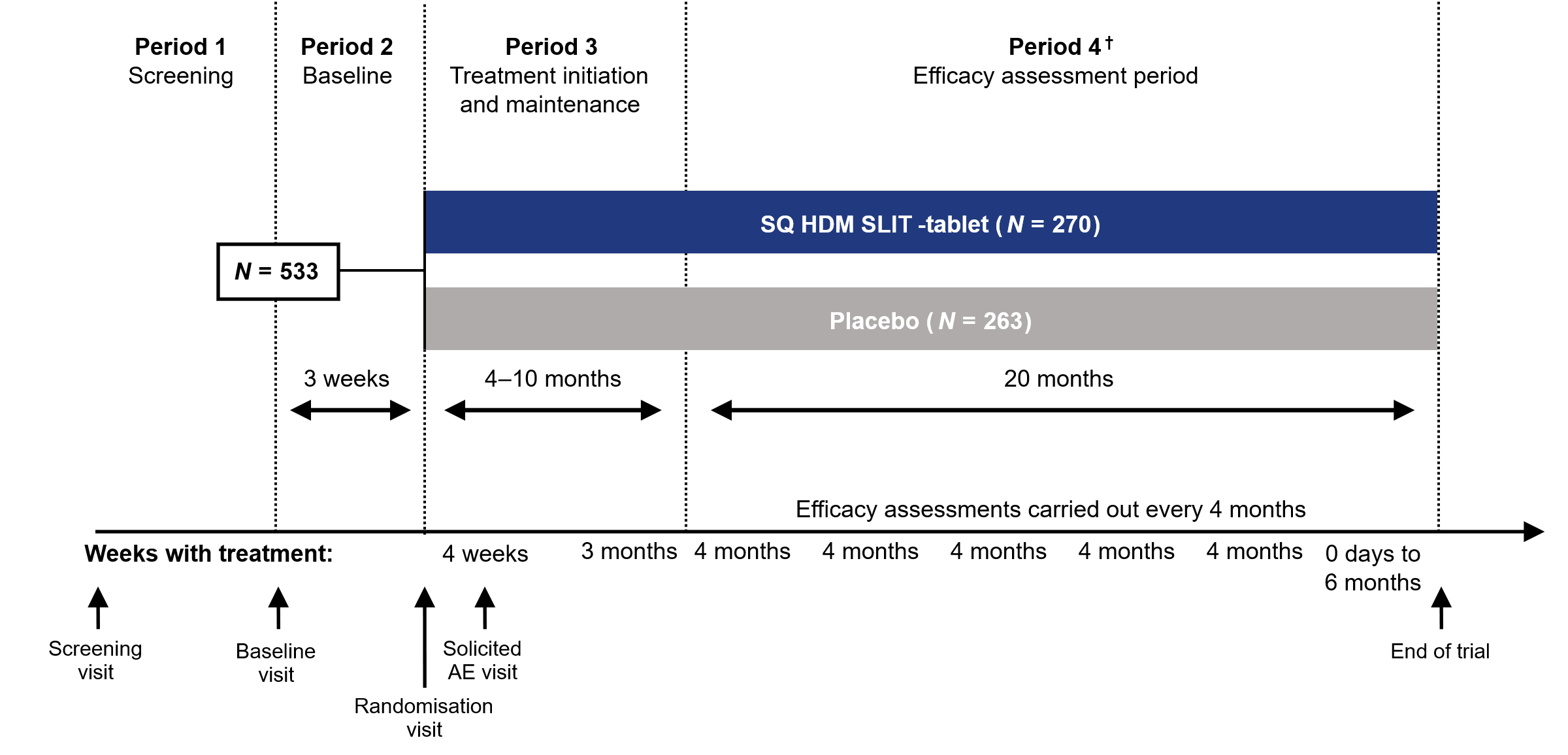
**Figure S1: Trial design**

The date of the first subject first visit was 22 February 2018, and of the last subject last visit was 31 May 2022. The trial ended in August 2022. ^†^The rate of clinically relevant asthma exacerbations was evaluated during the primary efficacy assessment period from 1 September to 30 April each year during the 2-year trial period. In total, subjects were treated for 24–30 months.

Abbreviations: AE=adverse event; HDM=house dust mite; SLIT=sublingual immunotherapy

**Trial population**

The inclusion and exclusion criteria are presented in the table below.

**Table S1: Inclusion and exclusion criteria**

| **Inclusion criteria** | |
| --- | --- |
| **Code** | **Criteria** |
| I1 | Written informed consent obtained from parents/caregivers before any trial-related procedures were performed. Consent or assent from the subject had to be obtained according to national requirements |
| I2 | Male or female of any race/ethnicity aged ≥4 to ≤17 years on the day informed consent was obtained from the parent/caregiver. The subject had to be ≥5 to ≤17 years old at the randomisation visit |
| I3 | A female subject of childbearing potential had to have a negative pregnancy test and be willing to practise appropriate contraceptive methods until the follow-up telephone call |
| I4 | A clinical history of HDM allergic asthma of ≥1 year duration diagnosed by a physician |
| I5 | Use of low daily dose of ICS plus LABA or medium/high daily dose of ICS with or without LABA for the control of asthma symptoms within the past year prior to randomisation |
| I6 | ≥ 3 clinically relevant asthma exacerbations in the past 2 years or ≥ 2 clinically relevant  asthma exacerbations in the past year or ≥1 severe asthma exacerbation in the past year  prior to randomisation while being on asthma controller medication (low dose ICS plus LABA or medium/high dose ICS with or without LABA).^†^ The asthma controller medication at the screening visit had to be at a dose equivalent to or below the dose the subject received before the last asthma exacerbation occurred |
| I7 | One or more of the following within the past 4 weeks prior to randomisation:  a) daytime asthma symptoms more than twice/week  b) any nocturnal awakening due to asthma, which required use of SABA rescue medication  c) SABA rescue medication needed for treatment of asthma symptoms more than twice/week  d) any activity limitation due to asthma |
| I8 | Lung function measured by FEV_1_ ≥70% of predicted value or according to local requirements while on controller medication following at least a 6-hour washout of SABA at screening and randomisation |
| I9 | Clinical history of HDM allergic rhinitis within the last year prior to randomisation |
| I10 | An average TCRS >0 during the baseline period (i.e., subjects were not required to meet a particular threshold for AR symptoms or to require AR symptom-relieving medication during the baseline period) |
| I11 | Positive specific IgE defined as ≥ class 2 (≥ 0.70 kU/l) against *D. pteronyssinus* and/or *D. farinae* at screening |
| I12 | Positive skin prick test to *D. pteronyssinus* and/or *D. farinae* at screening |
| I13 | Subject were willing and able to comply with trial protocol |
| **Exclusion criteria** | |
| E1 | Had a clinically relevant history and was sensitised, symptomatic, and regularly exposed to animal dander, moulds, and/or cockroach (e.g., present in the home, job, school, etc.) or another perennial allergen |
| E2 | Had experienced a life-threatening asthma attack defined in the protocol as an asthma episode that required intubation and/or was associated with hypercapnia requiring non-invasive ventilator support |
| E3 | Within the last month before the randomisation visit (Visit 3), had had an occurrence of any clinical deterioration of asthma that resulted in emergency treatment, hospitalisation, or treatment with systemic corticosteroids |
| E4 | Within the last 3 months before the randomisation visit (Visit 3) while on high dose ICS treatment, had had an occurrence of any clinical deterioration of asthma that resulted in emergency treatment, hospitalisation, or treatment with systemic corticosteroids |
| E5 | SLIT treatment with *D. pteronyssinus* or *D. farinae* for more than 1 month within the last 5 years. In addition, any SLIT treatment with *D. pteronyssinus* or *D. farinae* within the previous 12 months |
| E6 | SCIT treatment with *D. pteronyssinus* or *D. farinae* reaching the maintenance dose within the last 5 years. In addition, any SCIT treatment with *D. pteronyssinus* or *D. farinae* within the previous 12 months |
| E7 | Ongoing treatment with any allergy immunotherapy product |
| E8 | Severe chronic oral inflammation |
| E9 | Any nasal or naso/oropharyngeal condition that could confound the efficacy or safety assessments (e.g., hypertrophy of the pharyngeal/palatine tonsils, clinically relevant nasal polyps, a history of paranasal sinus surgery or surgery of nasal turbinates) |
| E10 | Any clinically relevant chronic disease including malignancy that in the opinion of the investigator would interfere with the trial evaluations or the safety of the subject |
| E11 | Had a diagnosis or history of eosinophilic oesophagitis |
| E12 | A relevant history of systemic allergic reaction, e.g., anaphylaxis with cardiorespiratory symptoms, generalised urticaria or severe facial angioedema that in the opinion of the investigator may constitute an increased safety concern |
| E13 | Active or poorly controlled autoimmune diseases, immune defects, immunodeficiencies, immunosuppression or malignant neoplastic diseases with current disease relevance |
| E14 | Ongoing treatment with oral corticosteroids |
| E15 | Treatment with restricted and prohibited concomitant medication |
| E16 | Treatment with an investigational drug within 30 days/5 half-lives of the drug (which ever longest) prior to screening |
| E17 | A history of allergy, hypersensitivity or intolerance to any of the excipients or active substance of the investigational medicinal product (except *D. pteronyssinus* and *D. farinae*) or to any excipient of the rescue medication provided in this trial |
| E18 | A business or personal relationship with trial or sponsor staff who was directly involved with the conduct of the trial |
| E19 | A history of alcohol or drug abuse |
| E20 | Had previously been randomised into this trial, was participating in this trial at another investigational site or was participating or planning to participate in any other clinical trial during the duration of this trial |
| E21 | Had a history or current evidence of any condition, treatment, laboratory values out of range or other circumstance that in the opinion of the investigator are clinically relevant and might expose the subject to risk by participating in the trial, confound the results of the trial, or interfere with the subject’s participation for the full duration of the trial |
| E22 | Had a condition or treatment that increases the risk of the subject developing severe adverse reactions after adrenaline administration |
| E23 | Has a condition or requires treatment that may increase the risk of the subject developing severe adverse reactions after adrenaline/epinephrine administration |
| E24 | Was unable to or would not comply with the use of adrenaline autoinjectors for countries where this was a regulatory requirement |

^†^A severe asthma exacerbation was defined as: systemic corticosteroids use for asthma symptom treatment for ≥3 days; or an emergency room visit because of asthma requiring treatment with systemic corticosteroids; or hospitalisation (admission not required) for >12 hours because of asthma. A clinically relevant asthma exacerbation was defined using the aforementioned criteria, with one additional criterion: doubling of ICS dose compared to controller medication. The primary efficacy endpoint definition for a clinically relevant asthma exacerbation was determined based on regulatory guidelines for asthma, as well as the joint statement on endpoints for clinical asthma trials and clinical practice from the American Thoracic Society and the European Respiratory Society.^1^

Abbreviations: AR=allergic rhinitis; HDM=house dust mite; ICS=inhaled corticosteroids; IgE=immunoglobulin type E; LABA=long-acting β2 agonist; FEV_1_=forced expiratory volume in second; SABA=short-acting beta-2 agonist; SCIT=subcutaneous immunotherapy; SLIT=sublingual immunotherapy; TCRS=total combined rhinitis symptom score.

**Randomisation and blinding**

The randomisation list was generated by a trial-independent statistician using interactive response technology; no stratification was used. Subjects, site personnel, and trial sponsor personnel were blinded to treatment; only members of the Data Monitoring Committee were unblinded.

**Intervention medication**

The dose tested was 12 SQ-HDM; SQ is a method for standardisation on biological potency, major allergen content and complexity of the allergen extract.

Subjects were provided with asthma rescue medication, including reliever medication (SABA, inhaler 100 μg/dose) and treatment for asthma exacerbations (budesonide, inhaler 100 and/or 200 μg/dose; fluticasone propionate, inhaler 50 μg/dose; and prednisolone/prednisone tablets, 5 mg), for use as needed. Use of prednisone tablets or doubling of ICS dose (budesonide or fluticasone inhaler) versus controller treatment was permitted only under investigator supervision.

Throughout the trial, subjects were expected to continue with the same asthma controller treatment (low dose inhaled ICS plus LABA, or medium/high dose ICS with or without LABA) as before entering the trial.

**Asthma controller medication**

The SQ HDM SLIT-tablet was administered as an add-on to controller medication. The controller medication was defined as either low dose inhaled ICS plus LABA, or medium/high dose ICS with or without LABA. Subjects were required to maintain their asthma controller medication throughout the trial.

Asthma severity was assessed based on daily ICS dose at baseline (low, medium, or high dose) with pre-defined thresholds for subjects 5–11 years old and ≥12 years of age (Table S2).

**Table S2: Definition of low, medium, and high dose ICS according to subject age**

| **ICS** | **Low daily dose (μg)** | **Medium daily dose (μg)** | **High daily dose (μg)** |
| --- | --- | --- | --- |
| **5–11 years** | | | |
| Beclomethasone dipropionate | 100–200 | >200–400 | >400 |
| Budesonide DPI | 100–200 | >200–400 | >400 |
| Budesonide nebuliser | 250–500 | >500–1,000 | >1,000 |
| Flunisolide | 500–750 | >750–1,250 | >1,250 |
| Fluticasone propionate | 100–200 | >200–500 | >500 |
| Ciclesonide | 80–160 | >160–320 | >320 |
| Mometasone furoate | 100 | 200 | >200 |
| Triamcinolone acetonide | 400–800 | >800–1,200 | >1,200 |
| **≥12 years** | | | |
| Beclomethasone dipropionate | 200–500 | >500–1,000 | >1,000 |
| Budesonide DPI | 200–400 | >400–800 | >800 |
| Flunisolide | 500–1,000 | >1,000–2,000 | >2,000 |
| Flunisolide furoate | 100 | NA | 200 |
| Fluticasone propionate | 100–250 | >250–500 | >500 |
| Ciclesonide | 80–160 | >160–320 | >320 |
| Mometasone furoate | ≥200 | ≥400 | >400 |
| Triamcinolone acetonide | 400–1,000 | >1,000–2,000 | >2,000 |

Information is based on guidance from the Global Initiative for Asthma (GINA) 2017 report and the National Asthma Education Prevention Program (NAEPP) Expert Panel Report 2007.^2,3^

Abbreviations: DPI=dry powder inhaler; ICS=inhaled corticosteroid; NA=not applicable

**Impact of the COVID-19 pandemic**

Due to the outbreak of the COVID-19 pandemic in early 2020, screening and randomisation were paused in March 2020, with all subjects in the screening phase as of 20 March 2020 recorded as screening failures. For subjects who continued in the trial during the pandemic, planned onsite visits were conducted remotely, and onsite trial monitoring visits were conducted remotely or were cancelled.

An additional recruitment cohort was planned, but not initiated due to the continuing COVID-19 pandemic, during which it was difficult to recruit subjects with a recent history of asthma exacerbations due to the decreasing asthma exacerbation rate observed in the general population.^4,5^ The annualised rate of clinically relevant asthma exacerbations decreased from 0.39 before the COVID-19 pandemic to 0.13 during the pandemic in the placebo group, and from 0.38 to 0.12 in the SQ HDM SLIT-tablet group. Due to the low asthma exacerbation rate during the COVID-19 pandemic impacting trial recruitment, the sponsor (ALK-Abellό A/S) decided to end the trial earlier than planned, on 10 August 2022, without further recruitment.

**Trial endpoints**

**Global evaluation of asthma.** At the final visit, subjects were asked how they felt when compared to their asthma before entering the trial, on a 5-point scale where 1=much better and 5=much worse. A subject answering ‘better’ or ‘much better’ was categorised as having ‘improved’ allergic asthma and a subject answering ‘the same’, ‘worse’, or ‘much worse’ was categorised as having allergic asthma that was ‘not improved’.

**Asthma control.** To evaluate each subject’s asthma control, ACQ and ACQ-IA total scores were measured every 4 months during the efficacy assessment period. For subjects aged 5–10 years, the interview-based ACQ-IA was used. The ACQ-IA has only been validated for children aged 6 years old;^6^ however, as this was a 2-year trial, it was used for subjects aged 5 years old given that they would be 7 years old by the end of the trial. For subjects aged ≥11 years, the self-completed ACQ was used – if this was not possible, the interview-based ACQ-IA was used. Both questionnaires comprised five scoring symptoms, as well as one question on SABA medication use, and one question on FEV_1_ (the latter was completed by clinic staff). Subjects were asked to recall their experiences during the previous 7 days and respond to each question using a 7-point scale, ranging from 0=well controlled to 6=extremely poorly controlled. The ACQ/ACQ-IA total score was calculated as the mean of the seven equally weighted items. Due to missing measurements of FEV_1_ caused by the COVID-19 pandemic, ACQ/ACQ-IA data were analysed without the FEV_1_ measurement.

**Immunology.** Blood samples for immunology assessments were taken at screening and at 12 and 24–30 months after randomisation. HDM-specific IgE, IgG_4_, and IgE-BF against *D. pteronyssinus* and *D. farinae* were measured.

**Safety assessment.** AEs, whether reported by the subject, detected through physical examination, laboratory test or other means, were recorded from the first trial-related activity until the last follow-up contact. AEs and serious adverse events (SAEs) were coded by qualified personnel using the Medical Dictionary for Regulatory Activities (MedDRA), version 20.1. Regulatory authorities and independent ethics committees/institutional review boards were informed of SAEs in accordance with local requirements and ICH guidelines for Good Clinical Practice.

Selected AEs were considered AESIs, which were: systemic allergic reactions including anaphylaxis; events treated with adrenaline/epinephrine; severe local swelling or oedema of the mouth and/or throat; oral allergy syndrome; eosinophilic oesophagitis; severe asthma exacerbations and clinically relevant asthma exacerbations.

Safety assessments included TRAEs (defined as TEAEs reported as ‘possibly’ related to trial medication by the investigator) and treatment-emergent asthma-related events. Asthma-related events were identified using the standardised MedDRA term, ‘Asthma/bronchospasm’.

**Statistical analysis**

**Sample size.** To determine sample size for the trial, power calculations were performed with two different distributions: Poisson distribution assuming no overdispersion (overdispersion means that some subjects will have more exacerbations than others) and negative binomial distribution assuming overdispersion (the dispersion parameter was set at k=0.7). Assuming an annual rate of 1.4 clinically relevant asthma exacerbations per subject in the placebo group,^7^ a 30% dropout rate, and an efficacy assessment period of 20 months (86 weeks), the trial planned to randomise 600 subjects (300 in each treatment arm) to ensure 90% power to detect superiority of treatment with SQ HDM SLIT-tablet versus placebo (20% between-group difference without overdispersion; 30% between-group difference in case of overdispersion). The expected annual event rate of clinically relevant asthma exacerbations per subject was calculated using the rate observed over 24 weeks in the placebo group of a randomised, double-blind trial by Lanier et al. (2009), which recruited children aged 6–11 years who had perennial allergen sensitivity and a history of asthma exacerbations and symptoms despite treatment with medium/high doses of ICS (with or without other controller medications).^7^ The rate of clinically relevant asthma exacerbations over 24 weeks in the trial reported by Lanier et al. (2009) was 0.64,^7^ which approximates to 1.4 per subject over 52 weeks.

**Full analysis set.** The FAS was defined as all randomised subjects who received at least one dose of trial medication and who were included according to their randomised treatment.

**Safety analysis set.** The safety analysis set was defined as all randomised subjects who received at least one dose of trial medication and who were included according to the treatment they received.

**Multiplicity adjustments.** To handle multiple testing of the primary and key secondary efficacy endpoints, hierarchical testing was employed on the FAS, primary estimand. The order of hypothesis testing was:

1. superiority testing of the SQ HDM SLIT-tablet over placebo with respect to the rate of clinically relevant asthma exacerbations during the efficacy evaluation period (primary endpoint)
2. superiority testing of the SQ HDM SLIT-tablet over placebo with respect to proportions of days with nocturnal awakenings due to asthma which require SABA use during the 14 days eDiary period recorded every 4 months after randomisation (first key secondary endpoint)
3. superiority testing of the SQ HDM SLIT-tablet over placebo with respect to the proportions of days with SABA use during the 14 days eDiary recording every 4 months after randomisation (second key secondary endpoint)
4. superiority testing of the SQ HDM SLIT-tablet over placebo with respect to the percentage predicted FEV_1_ assessed every 4 months after randomisation (third key secondary endpoint).

A lower test was to be evaluated only when the null hypothesis in the former test was rejected. Any test outside the test hierarchy was not adjusted for either multiplicity (type I error) or type II error, and observed p-values are presented.

**Other secondary endpoints.** Global evaluation of asthma was analysed using a generalised linear mixed model with logit link function, including treatment, age group (<12 years, ≥12 years), and region as fixed factors (presented as odds ratio with 95% CIs). ACQ/ACQ-IA data were analysed using an MMRM (presented as adjusted means with 95% CIs). Serum concentrations for HDM-specific IgE and IgG_4_, but not IgE-BF, were log10 transformed and the difference in the change from baseline between the SQ HDM SLIT-tablet and placebo was analysed using an MMRM (presented as adjusted means with 95% CIs).

**Results**

**Figure S2: Subject disposition**


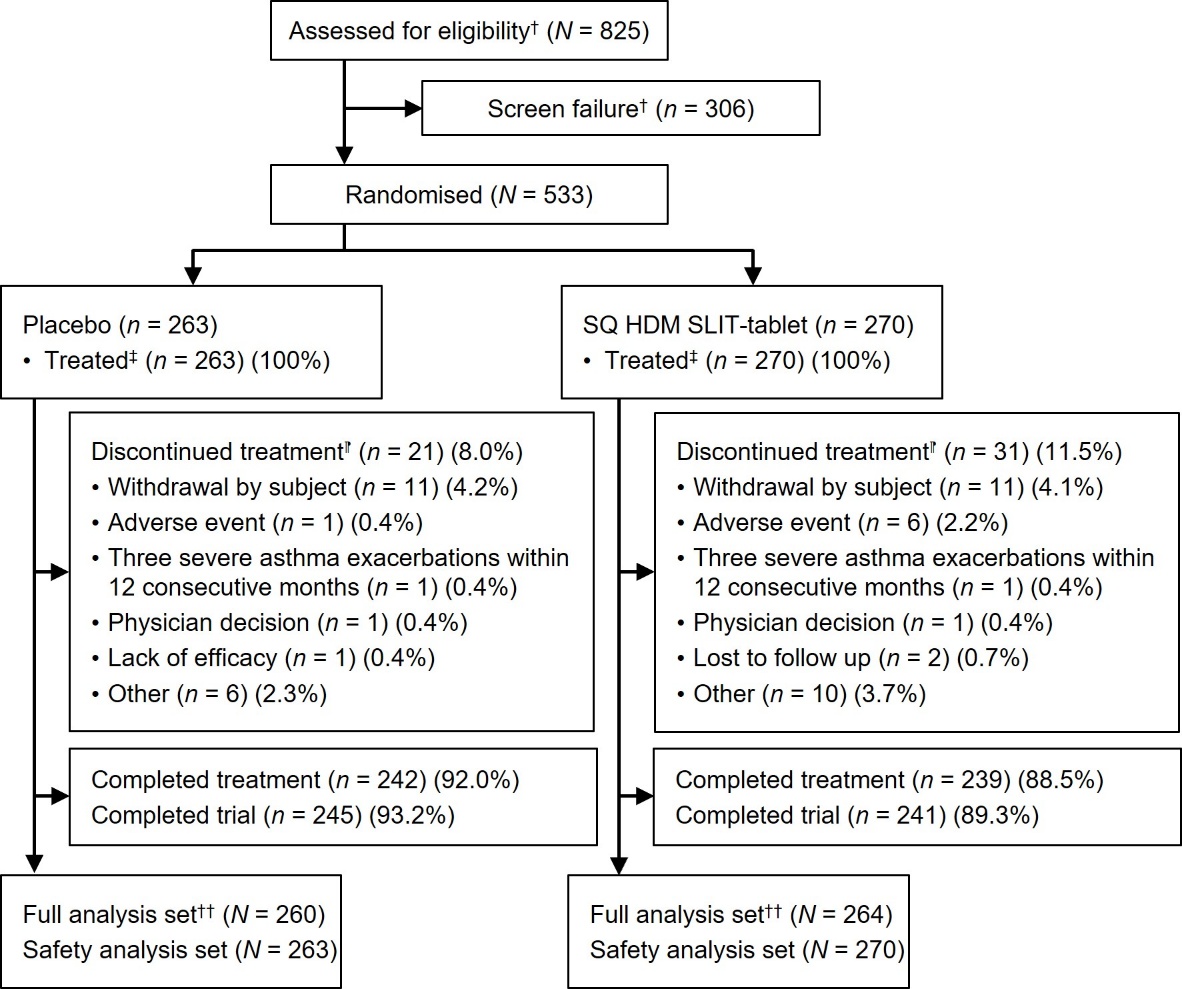


^†^Re-screened subjects were counted once as subjects screened and once as screen failures, whether or not they failed re-screening. ^‡^Subjects counted in the arm to which they were randomised. ^⁋^Includes discontinuing treatment for any reason, whether or not the subject discontinued the trial. ^††^Due to a breach of GCP at one trial site, all nine subjects from that site were excluded from the FAS.

Abbreviations: FAS=full analysis set; HDM=house dust mite; GCP=Good Clinical Practice; SLIT=sublingual immunotherapy


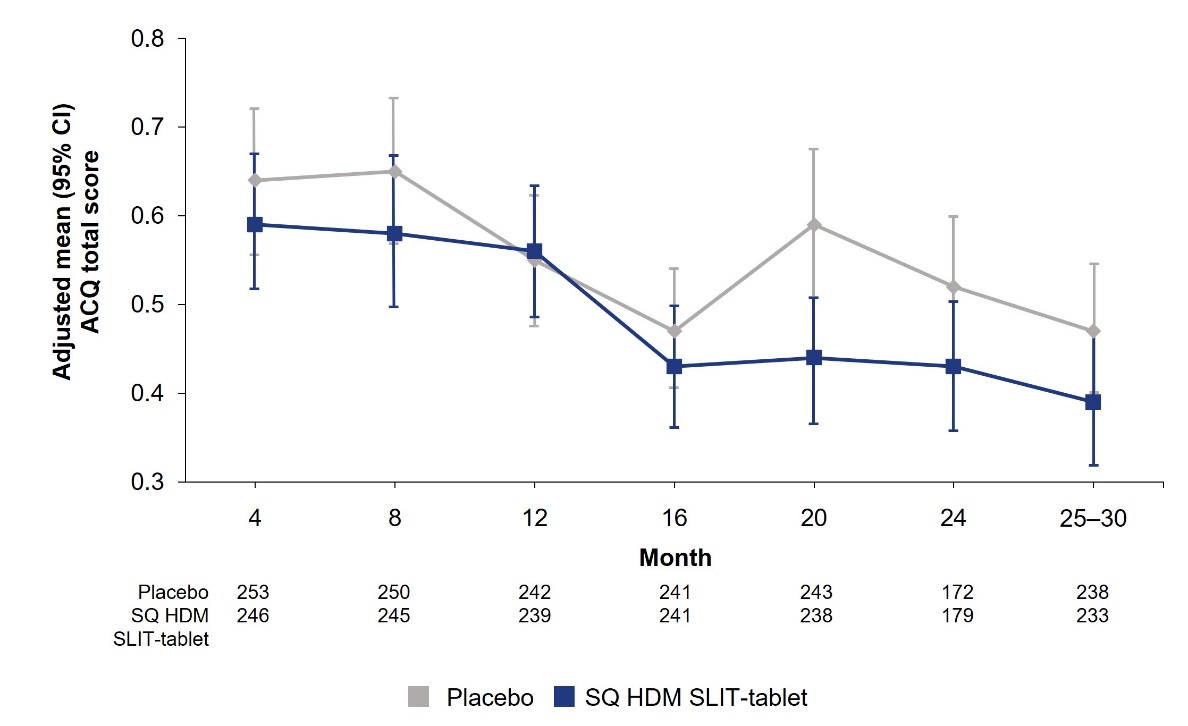
**Figure S3: ACQ total score excluding percentage-predicted FEV_1_ value (FAS)**

Numbers within the panel represent the number of subjects contributing to the adjusted means. Mean (SD) baseline ACQ total scores were: 1.2 (0.7) for placebo; 1.2 (0.8) for SQ HDM SLIT-tablet.

Abbreviations: ACQ=Asthma Control Questionnaire; CI=confidence interval; FAS=full analysis set; FEV_1_=forced expiratory volume in 1 second; HDM=house dust mite; SLIT=sublingual immunotherapy


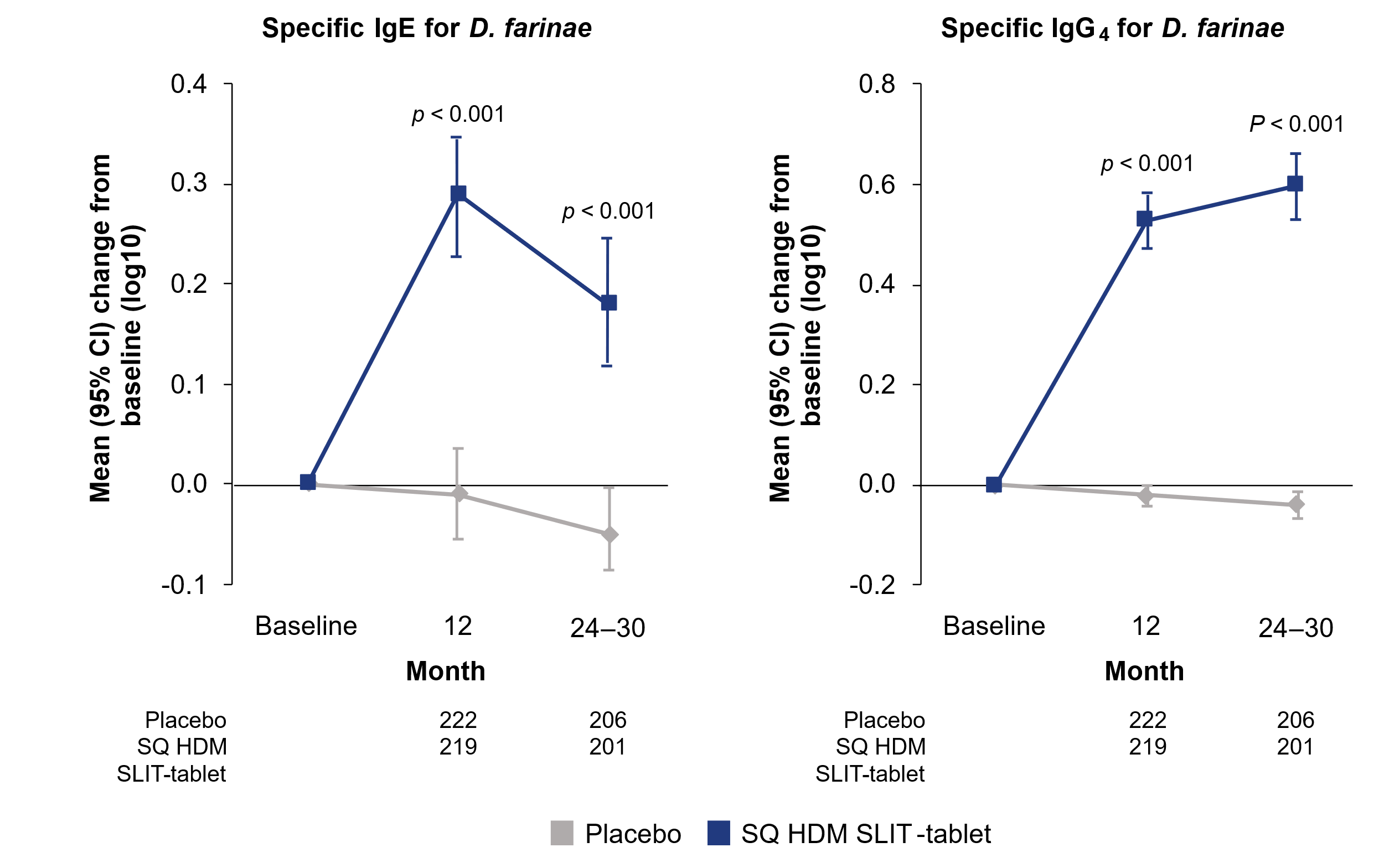
**Figure S4: Changes from baseline in immunological parameters – HDM-specific IgE and IgG_4_ against *D. farinae* (FAS)**

Numbers within the panel represent the number of subjects contributing to the adjusted means.

Abbreviations: CI=confidence interval; FAS=full analysis set; HDM=house dust mite; IgE=immunoglobulin type E; IgG_4_=immunoglobulin type G_4_; SLIT=sublingual immunotherapy

**Table S3: TRAEs reported in >10% of subjects in either treatment group (safety analysis set)**

| **TRAEs, n (%)** | **Placebo (*N* = 263)** | **SQ HDM SLIT-tablet (*N* = 270)** |
| --- | --- | --- |
| Oral pruritus | 58 (22.1) | 164 (60.7) |
| Throat irritation | 82 (31.2) | 153 (56.7) |
| Ear pruritus | 51 (19.4) | 98 (36.3) |
| Upper abdominal pain | 56 (21.3) | 87 (32.2) |
| Glossodynia | 16 (6.1) | 68 (25.2) |
| Lip swelling | 11 (4.2) | 68 (25.2) |
| Nausea | 34 (12.9) | 64 (23.7) |
| Mouth swelling | 7 (2.7) | 63 (23.3) |
| Pharyngeal oedema | 13 (4.9) | 55 (20.4) |
| Swollen tongue | 6 (2.3) | 51 (18.9) |
| Dysgeusia | 35 (13.3) | 47 (17.4) |
| Diarrhoea | 24 (9.1) | 29 (10.7) |

TRAEs are TEAEs reported as ‘possibly’ related to trial medication by the investigator.

Abbreviations: HDM=house dust mite; SLIT=sublingual immunotherapy; TEAE=treatment-emergent adverse event; TRAE=treatment-related adverse event

**Table S4: Onset and duration of the four most frequently reported TRAEs in either treatment group (Safety analysis set)**

|  | **Placebo (*N* = 263)** | | **SQ HDM SLIT-tablet (*N* = 270)** | |
| --- | --- | --- | --- | --- |
|  | Median  time to onset  (P5%–P95%) | Median duration  (P5%–P95%) | Median  time to onset  (P5%–P95%) | Median duration  (P5%–P95%) |
| Median time to onset and duration (days) | | | | |
| Ear pruritus | 2 (1–23)  (*n* = 51) | 1 (1–11)  (*n* = 51) | 2 (1–23)  (*n* = 98) | 1 (1–16)  (*n* = 98) |
| Oral pruritus | 3 (1–20)  (*n* = 58) | 1 (1–19)  (*n* = 58) | 2 (1–15)  (*n* = 164) | 1 (1–26)  (*n* = 163) |
| Throat irritation | 3 (1–20)  (*n* = 82) | 1 (1–12)  (*n* = 82) | 1 (1–19)  (*n* = 153) | 2 (1–24)  (*n* = 152) |
| Upper abdominal pain | 7 (1–23)  (*n* = 56) | 1 (1–5)  (*n* = 56) | 6 (1–25)  (*n* = 87) | 1 (1–13)  (*n* = 87) |
| Median time to onset and duration on the first day of trial medication (minutes) | | | | |
| Ear pruritus | 7.5 (2–190)  (*n* = 12) | 5 (3–45)  (*n* = 7) | 10 (0–600)  (*n* = 24) | 14 (5–32)  (*n* = 7) |
| Oral pruritus | 5 (1–140)  (*n* = 14) | 7.5 (4–20)  (*n* = 9) | 5 (0–300)  (*n* = 64) | 12 (3–30)  (*n* = 24) |
| Throat irritation | 14.5 (2–285)  (*n* = 15) | 15 (5–60)  (n = 9) | 5 (1–290)  (*n* = 58) | 20 (5–600)  (*n* = 26) |
| Upper abdominal pain | 203 (1–405)  (*n* = 2) | 120 (120–120)  (*n* = 1) | 92 (5–560)  (*n* = 7) | 30 (30–35)  (*n* = 3) |

TRAEs are TEAEs reported as ‘possibly’ related to trial medication by the investigator.

Abbreviations: HDM=house dust mite; P5%=5% percentile; P95%=95% percentile; SLIT=sublingual immunotherapy; TEAE=treatment-emergent adverse event; TRAE=treatment-related adverse event

**Narratives for subjects reporting severe or serious TRAEs**

Severe TRAEs were reported for four subjects in the SQ HDM SLIT-tablet group, and for one subject in the placebo group. One serious TRAE was reported in one subject who received the SQ HDM-SLIT tablet.

**Severe TRAEs**

**Subject 1:** a 6-year-old male receiving the SQ HDM SLIT-tablet experienced pharyngeal oedema (coded as an event of hypersensitivity by the investigator) at Day 14 after treatment initiation. Treatment with SQ HDM SLIT-tablet was discontinued, and the event resolved the same day.

**Subject 2:** a 12-year-old male receiving the SQ HDM SLIT-tablet experienced two events of both oral pruritus and ear pruritus at Days 2 and 10 after treatment initiation. On Day 2, there was no change in SQ HDM SLIT-tablet treatment, and the subject had recovered by the following day. On Day 10, treatment was discontinued, and the subject recovered the same day. The subject experienced throat irritation at Day 10 post-treatment initiation, which resolved following treatment discontinuation.

**Subject 3:** a 10-year-old male receiving the SQ HDM SLIT-tablet experienced an event of hypersensitivity at Day 8 following treatment initiation. Treatment with SQ HDM SLIT-tablet was interrupted and the event resolved the same day. For the full narrative of the hypersensitivity reaction, see page 15 (‘Narratives for subjects reporting AESIs’; Subject 1).

**Subject 4:** a 15-year-old female receiving the SQ HDM SLIT-tablet experienced two events of both lip pruritus and lip swelling at Days 20 and 26 following treatment initiation. Treatment with SQ HDM SLIT-tablet was unchanged for each event, which resolved the same day.

**Subject 5:** a 9-year-old male receiving placebo experienced atopic dermatitis at Day 815 after treatment initiation. The subject was treated with prednisone and recovered after 16 days; treatment with placebo was discontinued. Prior to the reported event of atopic dermatitis that led to treatment discontinuation, the subject experienced atopic dermatitis three times between Days 713–776 after the first intake of placebo; all instances were deemed unlikely related to the treatment by the investigator.

**Serious TRAEs**

**Subject 1:** a 10-year-old male receiving the SQ HDM SLIT-tablet experienced eosinophilic oesophagitis assessed as serious and of moderate severity at Day 31 after treatment initiation. Prior to the reported event of eosinophilic oesophagitis, the subject had experienced five non-serious episodes of vomiting and nausea over a 3-week period, which began 6 days after treatment initiation. Treatment with SQ HDM SLIT-tablet was discontinued 30 days after treatment initiation. The subject experienced vomiting again at Day 31 post-treatment initiation and was hospitalised 3 days later. A diagnosis of eosinophilic oesophagitis was confirmed in the hospital by histopathology of a supracardial oesophagus biopsy, which indicated eosinophil predominant inflammation (50 eosinophils per high-power filed). A 24-hour pH oesophageal metry found no evidence of gastro-oesophageal reflux disease. The subject was discharged after 6 days hospitalisation and treated with omeprazole 40 mg for 14 days and, thereafter, 20 mg for 3 months plus a diet adjusted to age. The subject was later treated with mometasone. The outcome of eosinophilic oesophagitis was reported as recovered by the investigator (based on a biopsy) 479 days after treatment initiation.

**Narratives for subjects reporting AESIs**

No TEAEs of anaphylaxis or anaphylactic reactions were reported by the investigator for any subject.

Eight subjects (four in each treatment group) reported a total of nine treatment-emergent serious AEs of clinically relevant asthma exacerbations (five events in the placebo group and four events in the SQ HDM SLIT-tablet group). Of these events, three in the placebo group and two in the SQ HDM SLIT-tablet group were reported as severe. All events were assessed as unlikely related to treatment by the investigator.

Two subjects experienced a non-serious, treatment-emergent systemic allergic reaction as reported by the investigator, which was coded as an event of hypersensitivity. Both subjects were in the SQ HDM SLIT-tablet group. The two events were assessed as possibly related to treatment by the investigator, and none led to discontinuation of treatment:

- **Subject 1:** a 10-year-old male receiving SQ HDM SLIT-tablet experienced a systemic allergic reaction (as assessed by the investigator) at Day 8 after treatment initiation with symptoms of oral itching, abdominal pain, and nausea. The subject was treated with desloratadine and recovered the same day. Treatment with the SQ HDM SLIT-tablet was temporarily interrupted. The event of hypersensitivity was assessed as non-serious and severe. Prior to the reported event of hypersensitivity, the subject experienced throat irritation, upper abdominal pain, oral pruritus, throat swelling, and mouth swelling after treatment initiation.
- **Subject 2:** a 16-year-old male receiving SQ HDM SLIT-tablet experienced a systemic allergic reaction (as assessed by the investigator) two minutes after the first dose with symptoms of itchy erythema on the left side of the neck, lip swelling, and tongue elevation. The subject was treated with desloratadine and recovered after 48 minutes. No action was taken with the SQ HDM SLIT-tablet treatment. The event of hypersensitivity was assessed as non-serious and mild.

**Table S5: Summary of AESIs (safety analysis set)**

| **AESIs** | **Placebo (*N* = 263)** | **SQ HDM SLIT-tablet (*N* = 270)** |
| --- | --- | --- |
| Serious AEs of clinically relevant asthma exacerbations (including severe asthma exacerbations) | 4 (1.5)^†^ | 4 (1.5)^†^ |
| Anaphylactic reactions or anaphylaxis | 0 (0) | 0 (0) |
| Systemic allergic reactions | 0 (0) | 2 (0.7)^‡^ |
| Events treated with adrenaline | 0 (0) | 0 (0) |
| Severe local swelling or oedema of the mouth or throat | 0 (0) | 1 (0.4)^‡^ |
| Eosinophilic oesophagitis | 0 (0) | 1 (0.4)^‡^ |

Data are n (%). ^†^The events were assessed as unlikely related to treatment by the investigator; ^‡^the events were assessed as ‘possibly’ related to treatment by the investigator.

Abbreviations: AE=adverse event; AESI=event of special interest; HDM=house dust mite; SLIT=sublingual immunotherapy

**References**

1. H. K. Reddel, D. R. Taylor, E. D. Bateman, et al., “American Thoracic Society/European Respiratory Society Task Force on Asthma Control and Exacerbations. An Official American Thoracic Society/European Respiratory Society Statement: Asthma Control and Exacerbations: Standardizing Endpoints for Clinical Asthma Trials and Clinical Practice,” *American Journal of Respiratory and Critical Care Medicine* 180, no.1 (2009): 59–99.

2. Global Initiative for Asthma, “Global Strategy for Asthma Management and Prevention” (2017), accessed March 11, 2025, https://ginasthma.org/wp-content/uploads/2017/02/wmsGINA-2017-main-report-final_V2.pdf.

3. National Asthma Education and Prevention Program, “Expert Panel Report 3: Guidelines for the Diagnosis and Management of Asthma,” in: Bethesda (MD): National Heart, Lung, and Blood Institute (US); 2007: https://www.ncbi.nlm.nih.gov/books/NBK7232/. Accessed 29 January 2025.

4. H. K. Reddel, L. B. Bacharier, E. D. Bateman, et al., “Global Initiative for Asthma Strategy 2021: Executive Summary and Rationale for Key Changes,” *Jounal of Allergy and Clinical Immunology. In Practice 10, no. 1S (*2022): S1–S18.

5. I. P. Skene and P. E. Pfeffer, “Improved Asthma Control During the COVID‐19 Pandemic: Are There Lessons to Be Learnt?,” *Thorax* 76, no. 9 (2021): 852–853.

6. E. F. Juniper, K. Gruffydd-Jones, S. Ward, and K. Svensson, “Asthma Control Questionnaire in Children: Validation, Measurement Properties, Interpretation,” *European Respiratory Journal 36, no. 6 (* 2010): 1410–1416.

7. B. Lanier, T. Bridges, M. Kulus, A. F. Taylor, I. Berhane, and C. F. Vidaurre, “Omalizumab for the Treatment of Exacerbations in Children With Inadequately Controlled Allergic (IgE‐Mediated) Asthma,” *Journal of Allergy and Clinical Immunology* 124, no. 6 (2009): 1210–1216.
